# Tin-sulfur based catalysts for acetylene hydrochlorination

**DOI:** 10.3906/kim-2010-36

**Published:** 2021-06-30

**Authors:** Yibo WU, Fuxiang LI, Xiaoqiang LUO, Gang TIAN, Yunxiao FENG, Yongjun HAN, Li WANG, Songmao CHU, Yunli CAO, Kesheng CAO, Xiaoming HU, Xuejun SHI, Songtian LI, Guoxv HE, Qingbin LI

**Affiliations:** 1 College of Chemistry and Environmental Engineering, Pingding Shan University, Pingding Shan China; 2 College of Chemistry and Chemical Engineering, Taiyuan University of Technology, Taiyuan China

**Keywords:** ulfur, tin-based catalysts, acetylene hydrochlorination

## Abstract

In the present work, tin-sulfur based catalysts were prepared using Na_2_SO_3_ and (CH_3_SO_3_)_2_Sn and were tested in acetylene hydrochlorination. Based on the analysis of experiments results, the acetylene conversion of (CH_3_SO_3_)_2_Sn/S@AC is still over 90%after a 50 h reaction, at the reaction conditions of T = 200 ^o^C, V_HCl_/V_C2H2 _= 1.1:1.0 and C_2_H_2_-GSHV = 15 h^–1^. According to the results of X-ray photoelectron spectroscopy (XPS), HCl adsorption experiments, and acetylene temperature programmed desorption (C_2_H_2_-TPD), it is reasonable to conclude that the interaction between Sn and S not only can retard the oxidation of Sn^2+^ in catalysts but also strengthen the reactant adsorption capacity of tin-based catalysts. Furthermore, results obtained from nitrogen adsorption/desorption and XPS proved that the CH_3_SO_3_- can effectively decrease the coke deposition of (CH_3_SO_3_)_2_Sn/AC and thus prolong the lifetime of (CH_3_SO_3_)_2_Sn/AC.

## 1. Introduction

Poly vinyl chloride (PVC) with its good wear-proof and chemical erode resistant properties has been implemented in many aspects of human life and industrial production. Furthermore, PVC is predominately synthesized through free radical polymerization reactions of the vinyl chloride monomer (VCM). Because of the resource feature of rich-coal in many developing countries (e.g., China), they mainly adopted the activated carbon-supported mercury chloride as catalysts, which catalyze the acetylene hydrochlorination to manufacture vinyl chloride [1–3]. However, the higher toxicity, easily sublimation, and mercury abatement worldwide has prompted many scholars to research a series of novel catalysts using the green method, for the sustainable development of acetylene hydrochlorination [4–6].

The higher intrinsic activity of gold based catalysts than that of HgCl_2_ catalysts for acetylene hydrochlorination was firstly reported by Hutchings et al., who also discovered that the deactivation of Au based catalysts originated from the reduction of Au^3+^ to Au^0^ and the aggregations of Au species in catalysts [7–12]. However, the fatal problem for gold based catalysts is high cost. Later on, this problem was solved through nonprecious metals, such as Cu [13–16] and Sn [17–22]. Li et al. reported that the acetylene conversion of phosphorus-doped copper-based catalysts decreases from 99.0% to 97.2% after a 82 h reaction (reaction conditions: GHSV-C_2_H_2 _= 30 h^–1^, T = 140 ^o^C, V_HCl_/V_C2H2 _= 1.15:1.0) [13]. Due to the introduction of phosphorus into the catalysts, which can effectively not only improve the dispersion but also lessen the aggregation of copper species, Cu-P/AC features better catalytic performance than the Cu/AC catalyst in acetylene hydrochlorination [13]. Additionally, when the reaction condition was maintained at GHSV-C_2_H_2 _= 50 h^–1^ and T = 200 ^o^C, Zhai et al. founded that carbon supported CsCuCl_3_ as catalysts still exhibited 92% hydrochlorination activity over 200 h [14]. Recently, Ren et al. reported that the (methoxymethyl) triphenylphosphonium chloride (MOMTPPC) ionic liquid has a positive effect on the dispersion and stabilization of Cu species in catalysts [16]. Specifically, Cu@MOMTPPC/AC still reached the 98.7% acetylene conversion after the 360 h stability test under the conditions of GHSV-C_2_H_2 _= 36 h^–1^, T = 180 ^o^C, V_HCl_/V_C2H2 _= 1.2:1 [16]. Except for Cu catalysts, Deng et al. prepared SnCl_2_-BiCl_3_-CuCl/AC that can catalyze acetylene hydrochlorination and found that the deactivation of catalysts mainly attributed to the loss of tin(IV) chloride in acetylene hydrochlorination [17]. Although the tin-based catalysts exhibited considerable activity in acetylene hydrochlorination [18–20], the stability as the drawback is significant enough to restrict further development. In our previous study, it was discovered that the Sn^2+^ species is easily oxidized to Sn^4+^, whichresults in the loss of Sn species during catalysis of acetylene hydrochlorination [18,19]. Tai et al. found that the interaction between the sulfonate group and Sn^2+^ can stabilize the Sn^2+ ^and possibly retard the oxidation of Sn^2+ ^[21]. Additionally, Wang et al. reported that the doping of S can not only boost the specific surface areas but also decrease the active species particle size for the Bi-based catalysts [22]. 

In this work, Sn-based catalysts were prepared using Na_2_SO_3_ and (CH_3_SO_3_)_2_Sn, as well as tested in acetylene hydrochlorination. Through the characteristic techniques including BET, XRD, XPS, C_2_H_2_-TPD, HCl adsorption experiments and TG, the physicochemical properties and deactivation reason of Sn-based catalysts in the hydrochlorination of acetylene were thoroughly investigated. 

## 2. Experimental

### 2.1. Materials

Coal based columnar activated carbon was purchased from Shanxi Xinhua Activated Carbon Co. Ltd (Shanxi, China). (CH_3_SO_3_)_2_Sn, Na_2_CO_3_, Na_2_SO_3_, and C₁₄H₁₄N₃SO₃Na were purchased from Tianjin Kemiou Chemical Reagent Co. Ltd (Tianjin, China). C_2_H_2_ and HCl (99.9%) were purchased from Nanjing Shangyuan industrial gases Co. Ltd (Nanjing, China) and Dalian Special gases Co. Ltd (Liaoning, China), respectively.

### 2.2. Catalysts preparation 

Coal based columnar activated carbon was pretreated by 0.01 mol L^–1^ HCl to remove the ash in mirco- and mesopores. The obtained sample was dried at 100 ^o^C overnight and then denoted as AC.

1.0 g Na_2_SO_3_ was absolutely dissolved in 20 mL distill water, and then 9.0 g AC was added into the above solution and stirred at 100^ o^C for 1 h. The obtained heterogeneous solid was dried at 100^ o^C for 12 h, then calcined at 600 ^o^C under a N_2_ atmosphere for 5 h. The final samples were named as 10%S@AC. 

(CH_3_SO_3_)_2_Sn/S@AC was synthesized by equivalent-volumetric impregnation methods. Specifically, 0.4 g (CH_3_SO_3_)_2_Sn was mixed with an appropriate volume of ethanol and 3.6 g 10%S@AC and then stirred at for 4 h. The final samples was dried at 100 ℃ for 12 h, and the obtained catalysts was labeled as 10%(CH_3_SO_3_)_2_Sn/10%S@AC.

### 2.3. Catalysts characterization

To analyze the pore textural properties of fresh- and used catalysts, BET specific surface area, pore size distribution, and pore volume analysis was performed on Quantachrome Nova 2000e instruments (activation condition: T = 150^ o^C, time = 4 h). Through the X-ray diffraction (LabX XRD-6000, Shimadzu, city, country?), the dispersion of Sn species on the catalysts surface was discussed. Acetylene temperature-programmed desorption (C_2_H_2_-TPD) was carried out on FINESORB-3010 (Zhejiang Finetec Instruments Co. Ltd., Zhejiang, China). Before starting the test, a sample (50 mg) was treated with Ar atmosphere at 200^ o^C for 1 h, and then temperature was cooled to 25^ o^C in Ar atmosphere and a flow rate of 25 mL min^–1^. For C_2_H_2_ desorption experiments, the temperature was heated from 25 ^o^C to 500^ o^C (10^ o^C min^–1^) under Ar atmosphere (25 mL min^-1^). HCl adsorption/desorption experiments were performed on the fix-bed reactor. After the HCl adsorbed procedure, the reactor temperature increased from room temperature to 400^ o^C in an Ar atmosphere with a flow rate of 20 mL min^–1^. At the same time, the desorption gas was adsorbed by 1000 mL distill water. Furthermore, the hydrogen chloride adsorption capacity of catalyst was calculated by the acid-based titration method. In order to investigate the chemical state of Sn species and the interaction between Sn and S in catalysts, we adopted X-ray photoelectron spectroscopy (XPS, Escalab250i, Thermo Fisher Scientific, Waltham, MA, USA) techniques.

### 2.4. Catalytic test 

Hydrochlorination activity of catalysts (4.0 mL) was performed in fixed bed reactor (i.d =10 mm). An effective way to get rid of the air in lines and activate catalysts is to initially feed HCl into the reactor system. Later on, the temperature of reactor was maintained at 200 ^o^C using a temperature controller (Yudian, Hong Kong, China). When the GHSV-C_2_H_2_ and reaction temperature reaches 30 h^–1^ and 200^ º^C, respectively, the mole ratio of HCl and C_2_H_2_ was controlled at 1.1:1.0 by a mass flowmeter. When a dynamic catalytic acetylene hydrochlorination reaction was established, the product mixture gas was led into the adsorbed reactor (soda lime) to remove the unreacted HCl and consequently the cleaned gas was analyzed by GC900 instruments.

## 3. Results and discussion

### 3.1. Catalytic performance 

**Figure 1:**
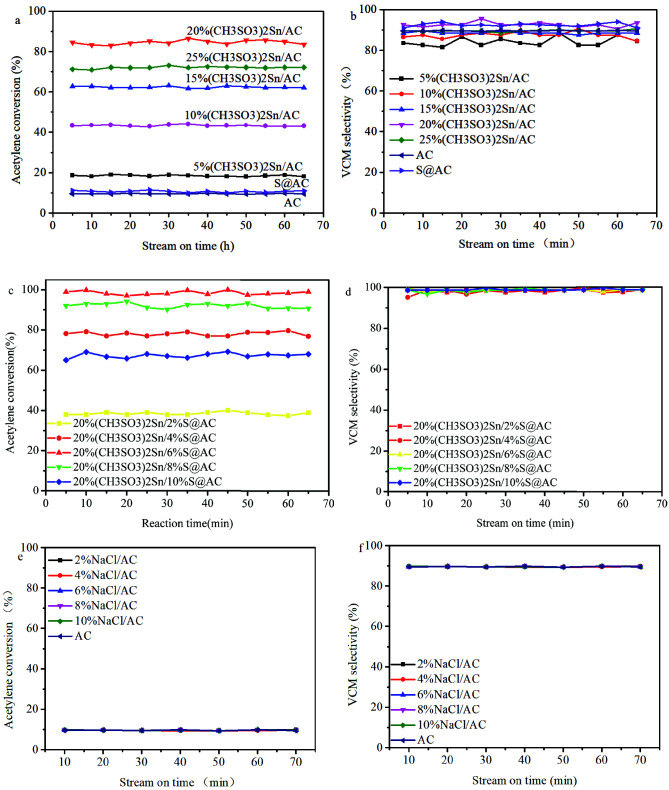
Catalytic activity of (a) (CH3SO3)2Sn/AC and (b) (CH3SO3)2Sn/S@AC, VCM selectivity of (c) (CH3SO3)2Sn/AC and (d) (CH3SO3)2Sn/S@AC (e) Catalytic activity of NaCl/AC; (f) VCM selectivity of NaCl/AC (reaction conditions: T = 200 ℃, C2H2-GHSV = 30 h-1 and VHCl/VC2H2 = 1.1/1.0).

The catalytic performance of (CH_3_SO_3_)_2_Sn/AC was related to the loading amount of (CH_3_SO_3_)_2_Sn, as shown in Figure 1a. Results summarize that when the loading amount of (CH_3_SO_3_)_2_Sn in AC increases from 5% to 25%, the initial C_2_H_2_ conversion of (CH_3_SO_3_)_2_Sn/AC increases from 18.7% to the maximum value of 85.1%, and then decreases to 71.9%. When the mole ratio of V_HCl_/V_C2H2_ is 1.1/1.0, the product composition of reaction by-products is 1,2-dichloroethane. Moreover, in the catalytic acetylene hydrochlorination (Figure 1b), VCM selectivity of catalysts was achieved in the following order: 20%(CH_3_SO_3_)_2_Sn/AC(95.5%) > 25%(CH_3_SO_3_)_2_/AC(90.5%) > 10%(CH_3_SO_3_)_2_Sn/AC(90.3%) > 15%(CH_3_SO_3_)_2_Sn/AC(89.5%) > 5%(CH_3_SO_3_)_2_Sn/AC(88.5%) (Table 1). On the basis of Figures 1a and 1b, for AC, it shows that Na_2_SO_3 _additives can lead to the increase of VCM selectivity but without a marked increase of acetylene conversion. Additionally, when loading of (CH_3_SO_3_)_2_Sn into AC, (CH_3_SO_3_)_2_Sn/AC features higher hydrochlorination activity when compared to AC and S@AC, suggesting that tin species are the main active sites in tin-sulfur based catalysts. Although (CH_3_SO_3_)_2_Sn/AC can catalyze the acetylene hydrochlorination, there was a gap compared with the activity and VCM selectivity of HgCl_2_/AC (Figure 1c). Consequently, the modification of (CH_3_SO_3_)_2_Sn/AC by Na_2_SO_3 _to improve the catalytic performance is based on the previous work [21–23]. As shown in Figure 1d, the VCM selectivity of 20%(CH_3_SO_3_)_2_Sn/2%S@AC, 20%(CH_3_SO_3_)_2_Sn/4%S@AC, 20%(CH_3_SO_3_)_2_Sn/6%S@AC, 20%(CH_3_SO_3_)_2_Sn/8%S@AC and 20%(CH_3_SO_3_)_2_Sn/10%S@AC is reached at 99.3%, 99.0%, 99.5%, 99.7%, and 99.7%, respectively (Table 1). Among these catalysts, the C_2_H_2_ conversion of 20% (CH_3_SO_3_)_2_Sn/6% S@AC reaches the maximum value of 100%, besides its VCM selectivity is over 98.5% (Figure 1d). This suggests that the doping of Na_2_SO_3 _additives into carriers and the synergistic effect between Sn and S display an impact on the enhancement of catalytic performance in acetylene hydrochlorination. To eliminate the effect of Na specieson the catalytic performance of catalysts for acetylene hydrochlorination, we solely studied the hydrochlorination activity of NaCl/AC. As shown in Figure 1e, with the NaCl raised from 2% to 10%, acetylene conversion over NaCl/AC all reached 10.2%, which is close to the value of AC (9.8%). Moreover, NaCl has a small influence on the VCM selectivity of AC in acetylene hydrochlorination. The abovementioned results suggested that Na over the catalyst surface does not have a positive effect on the catalytic performance.

**Table 1:** Selectivity of catalysts.

Sample	VCM selectivity (%)(maximum value)	1,2 dichloroethane selectivity(%)
AC	89.6	10.4
S@AC	92.1	7.9
5%(CH3SO3)2Sn/AC	88.5	11.5
10%(CH3SO3)2Sn/AC	90.3	9.7
15%(CH3SO3)2Sn/AC	89.5	10.5
20%(CH3SO3)2Sn/AC	95.5	4.5
25%(CH3SO3)2Sn/AC	90.5	9.5
20%(CH3SO3)2Sn/2%S@AC	99.3	0.7
20%(CH3SO3)2Sn/4%S@AC	99.0	1.0
20%(CH3SO3)2Sn/6%S@AC	99.5	0.5
20%(CH3SO3)2Sn/8%S@AC	99.7	0.3
20%(CH3SO3)2Sn/10%S@AC	99.7	0.3
2%NaCl/AC	90.2	9.8
4%NaCl/AC	90.1	9.9
6%NaCl/AC	90.3	9.7
8%NaCl/AC	90.5	9.5
10%NaCl/AC	89.9	10.1

### 3.2. Characterization of catalysts

3.2.1. Physical characteristics of catalysts

**Figure 2:**
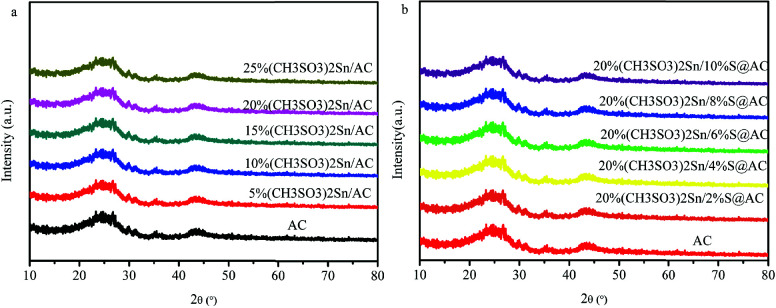
The XRD pattern of (a) (CH3SO3)2Sn/AC and (b) (CH3SO3)2Sn/S@AC.

As listed in Table 2, with increasing the loading amount of (CH_3_SO_3_)_2_Sn in AC, the specific surface area and total volume of catalysts decreased from 983 m^2^/g and 0.48 cm^3^/gto 262 m^2^/g and 0.14 cm^3^/g, respectively, suggesting that (CH_3_SO_3_)_2_Sn is successfully loaded into the carbon support. Furthermore, the specific surface area and total volume of 20%(CH_3_SO_3_)_2_Sn/S@AC is lower than that of 20%(CH_3_SO_3_)_2_Sn/AC (461 m^2^/g and 0.23 cm^3^/g), further demonstrating the introduction of Na_2_SO_3 _in 20%(CH_3_SO_3_)_2_Sn/AC (Table 3). Moreover, the presence of Sn species’ characteristic diffraction is not observed in (CH_3_SO_3_)_2_Sn-based catalysts, which all exhibit two discernable peaks of AC (Figures 2a and 2b). These results infer that active compounds and additives are well dispersed on the carbon surface. 

**Table 2:** Textual properties of (CH3SO3)2Sn/AC catalysts and AC.

Sample	SBET(m2/g)	Smicro(m2/g)	Smeso(m2/g)	Vtotal(cm3/g)	D(nm)
AC	983	854	129	0.48	1.9
5%(CH3SO3)2Sn/AC	806	730	76	0.32	1.8
10%(CH3SO3)2Sn/AC	628	580	48	0.27	1.7
15%(CH3SO3)2Sn/AC	544	446	98	0.25	2.0
20%(CH3SO3)2Sn/AC	461	389	72	0.23	2.1
25%(CH3SO3)2Sn/AC	262	213	48	0.14	2.3

**Table 3:** Textual properties of (CH3SO3)2Sn/S@AC catalysts.

Sample	SBET(m2/g)	Smicro(m2/g)	Smeso(m2/g)	Vtotal(cm3/g)	D(nm)
20%(CH3SO3)2Sn/AC	461	389	72	0.23	2.1
20%(CH3SO3)2Sn/2%S@AC	403	312	91	0.21	2.3
20%(CH3SO3)2Sn/4%S@AC	378	274	104	0.19	2.6
20%(CH3SO3)2Sn/6%S@AC	315	206	109	0.18	3.0
20%(CH3SO3)2Sn/8%S@AC	248	149	99	0.14	3.3
20%(CH3SO3)2Sn/10%S@AC	159	78	81	0.11	3.5

**Figure 3:**
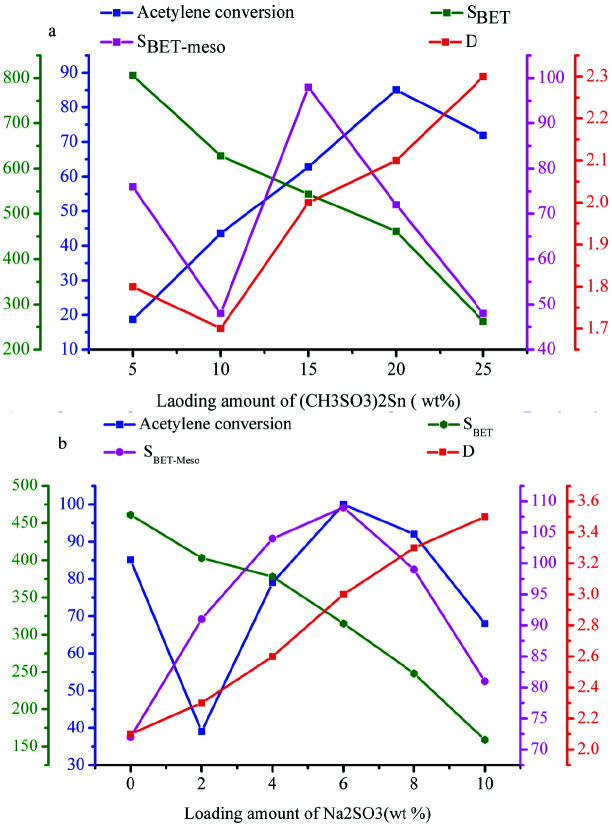
The correlation between the pore textual properties of catalysts and catalytic performance; (a) (CH3SO3)2Sn/AC and (b) (CH3SO3)2Sn/S@AC.

The effect of textural properties on the catalytic activity of catalysts was further studied by analyzing the relationship among the S_BET_-meso, S_BET_, average pore size, and acetylene conversion. As shown in Figure 3a, the acetylene conversion of (CH_3_SO_3_)_2_Sn/AC gradually increased with increases in the loading of (CH_3_SO_3_)_2_Sn from 5 wt% to 20 wt%, along with a decrease in the specific surface area of catalysts, suggesting that (CH_3_SO_3_)_2_Sn content is a main factor in the enhancement of catalytic performance. However, as the loading amount of (CH_3_SO_3_)_2_Sn was further increased from 20 wt% to 25 wt%, the specific surface area and acetylene conversion decreased, from 464 m^2^/g to 261 cm^2^/g and from 85.1% to 70.2%, respectively. This result suggests that the loss of specific surface area of catalysts directly leads to the decrease the acetylene conversion of (CH_3_SO_3_)_2_Sn/AC. The correlation between the textual properties and catalytic performance of (CH_3_SO_3_)_2_Sn/S@AC is shown in Figure 3b. The S_BET_-meso and acetylene conversion of catalysts features a similar tendency as the loading amount of Na_2_SO_3 _was increased. Significantly, the analysis results indicated that there is a strong positive correlation between acetylene conversion and S_BET_-meso.

#### 3.2.2. Chemical characteristics of catalysts

**Figure 4:**
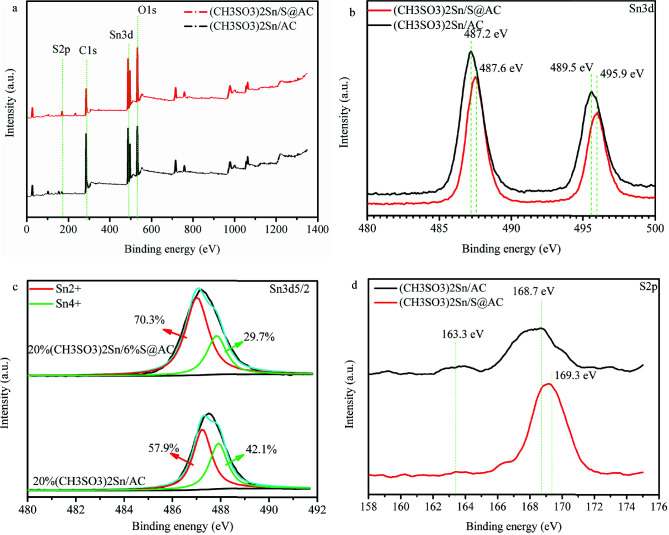
(a) XPS pattern of catalysts; (b) XPS-S2p pattern of catalysts; (c) XPS-Sn3d pattern of catalysts; (d) XPS-Sn3d5/2 pattern of catalysts.

X-ray photoelectron spectroscopy (XPS) was employed to study the elemental valence and chemical bond structure. Table 4 and Figure 4a prove the coexistence of Sn, S, O, C, and Na in catalysts. Furthermore, it can be observed in Figure 4b, that the two peaks at 487.2~487.6 eV and 489.5~495.9 eV correspond to Sn3d_3/2_and Sn3d_5/2_, respectively [24,25]. However, the Sn3d peaks over (CH_3_SO_3_)_2_Sn/AC exhibit a negative shift compared to (CH_3_SO_3_)_2_Sn/S@AC, showing the existence of an interaction between the S@AC and Sn species (Figure 4c). For comparison, Table 5 lists Sn species content in catalysts. The content of Sn^2+^ and Sn^4+^ in (CH_3_SO_3_)_2_Sn/AC reaches 1.61 wt% and 1.17 wt%, respectively. (CH_3_SO_3_)_2_Sn/S@AC demonstrates the higher Sn^2+^ content of 2.05 wt%, having an increase of 27.4% in comparison with (CH_3_SO_3_)_2_Sn/AC (1.61 wt%). The above mention results suggest that the synergistic effects between Na_2_SO_3_ and Sn species can lessen the oxidation of Sn^2+^. This result is in agreement with the previous study [21]. As shown in Figure 4d, two characteristic peaks were observed at 163.3eV and 168.7eV in (CH_3_SO_3_)_2_Sn/AC, which can be assigned as SO_4_^2-^ as well as SO_3_^2-^[26–28]. The main S2p peak shifted from 168.7eV to 169.3eV, confirming that the interaction between Sn species and S@AC existed in (CH_3_SO_3_)_2_Sn/S@AC. Na_2_SO_3_ pyrolysis can generate Na_2_S and Na_2_SO_4. _Combing Figure 1b and the above results, it is shown that Na_2_S and NaSO_4 _has a small effect on the catalytic activity. Additionally, Zhou et al *.* reported that (CH_3_SO_3_)_2_Sn can keep stable at a range of 100–390 ℃ and found that (CH_3_SO_3_)_2_Sn is decomposed into SnSO_4_ at 390–407 ℃ [29]. Based on the analysis of the above mentioned results,CH_3_SO_3_- displays a positive influence on the hydrochlorination activity of tin-based catalysts.

**Table 4:** Elements loading amount of catalysts (XPS).

Samples	Elements loading (wt%)				
	Sn	S	O	C	Na
(CH3SO3)2Sn/AC	2.78	1.71	27.17	67.81	0.53
(CH3SO3)2Sn/S@AC	2.92	5.17	37.04	54.07	0.80

**Table 5:** Sn species content of catalysts (XPS).

Samples	Elements loading (wt%)	
	Sn2+	Sn4+
(CH3SO3)2Sn/AC	1.61 (57.9%)	1.17 (42.1%)
(CH3SO3)2Sn/S@AC	2.05 (70.2%)	0.87 (29.8%)

The previous study demonstrated that the ability of adsorbing reactant (C_2_H_2_ and HCl) is related to active sites [30,31]. In Figure 5a, the desorption peak area of (CH_3_SO_3_)_2_Sn/S@AC was larger than that of (CH_3_SO_3_)_2_Sn/AC. As depicted in Figure 5b, HCl adsorption/desorption experiments (Tables 6–9) are used to calculate the HCl adsorption capacity of catalysts. Specifically, HCl adsorption capacity of AC, S@AC, (CH_3_SO_3_)_2_Sn/AC and (CH_3_SO_3_)_2_Sn/S@AC was found to be 0.18 mmol/g, 0.23 mmol/g, 0.27 mmol/g, and 0.31 mmol/g, respectively.The abovementioned results suggest that the main active sites are (CH_3_SO_3_)_2_Sn, and that the interaction between Sn species and S@AC can enhance the ability for reactant adsorption, resulting in the enhancement of catalytic activity.

**Table 6:** The mathematical parameters for calculating the HCl adsorption capacity of (CH3SO3)2Sn/S@AC.

Numbers	Na2CO3(mL)	Adsorption volume(mL)	Catalysts(g)	HCl adsorption capacity(mmol/g)
1	14,080	250	3.52	0.32
2	13,718	250	3.54	0.31
3	13,163	250	3.51	0.30
4	13679	250	3.53	0.31

**Table 7:** The mathematical parameters for calculating the HCl adsorption capacity of (CH3SO3)2Sn/AC.

Numbers	Na2CO3(mL)	Adsorption volume(mL)	Catalysts(g)	HCl adsorption capacity(mmol/g)
1	11,779	250	3.49	0.27
2	11,375	250	3.50	0.26
3	12,180	250	3.48	0.28
4	10,969	250	3.51	0.25

**Table 8:** The mathematical parameters for calculating the HCl adsorption capacity of S@AC.

Numbers	Na2CO3(mL)	Adsorption volume(mL)	Catalysts(g)	HCl adsorption capacity(mmol/g)
1	9861	250	3.43	0.23
2	8978	250	3.42	0.21
3	9890	250	3.44	0.23
4	10,290	250	3.43	0.24

**Table 9:** The mathematical parameters for calculating the HCl adsorption capacity of AC.

Numbers	Na2CO3(mL)	Adsorption volume(mL)	Catalysts(g)	HCl adsorption capacity(mmol/g)
1	7203	250	3.39	0.17
2	7225	250	3.40	0.17
3	7456	250	3.41	0.19
4	7650	250	3.40	0.18

### 3.3. Deactivation of catalysts

In order to evaluate the long-term stability experiments of tin-sulfur based catalysts, 20%(CH_3_SO_3_)_2_Sn/6%S@AC and 20%(CH_3_SO_3_)_2_Sn/AC are tested at GHSV = 15 h^–1^, respectively. As shown in Figure 6, the initial acetylene conversion of 20% (CH_3_SO_3_)_2_Sn/6% S@AC achieves 100%. Furthermore, it can be seen that the acetylene conversion was more than 90% after 50 h reaction. At same time, 20% (CH_3_SO_3_)_2_Sn/AC reaches only 22.8%. This results confirm that S additives can strengthen the durability of 20%(CH_3_SO_3_)_2_Sn/AC. Table 10 lists that the loss of specific surface area and pore volume of 20%(CH_3_SO_3_)_2_Sn/6%S@AC is 208 m^2^/g and 0.10 cm^3^/g, compared to 20%(CH_3_SO_3_)_2_Sn/AC (which is 345 m^2^/g, 0.13cm^3^/g), indicating that the deposition of carbonaceous material on the catalyst surface during acetylene hydrochlorination. Therefore, the coke deposition of catalysts is one of reasons for deactivation. The loss rate of specific surface area over used-20%(CH_3_SO_3_)_2_Sn/AC and used-20%(CH_3_SO_3_)_2_Sn/6%S@AC is 74.8% and 66.0%, respectively, which suggests that S additives can improve the anticoking ability and catalytic performance of (CH_3_SO_3_)_2_Sn based catalysts. Hutchings et al. firstly studied the Au-based catalysts, which feature the superior catalytic performance in acetylene hydrochlorination [7]. However, Au-based catalysts also have limitations. The major problem of Au-based catalysts is they are expensive compared to nonprecious metal catalysts ((CH_3_SO_3_)_2_Sn/S@AC).

**Table 10:** Textual properties of fresh- and used catalysts.

Sample	SBET(m2/g)	Smicro(m2/g)	Smeso(m2/g)	Vtotal(cm3/g)	D(nm)
Fresh-20%(CH3SO3)2Sn/AC	461	389	72	0.23	2.1
Used-20%(CH3SO3)2Sn/AC	116	93	23	0.10	2.4
Fresh-20%(CH3SO3)2Sn/6%S@AC	315	206	109	0.18	3.0
Used-20%(CH3SO3)2Sn/6%S@AC	107	61	46	0.08	3.2

## 4. Conclusion

Overall, the physical and chemical properties of (CH_3_SO_3_)_2_Sn/AC catalysts were studied by BET, XRD, XPS, C_2_H_2_-TPD, and HCl adsorption experiments. The S additives doped in AC can significantly and effectively decrease the coke deposition of (CH_3_SO_3_)_2_Sn/AC, therefore strengthening the reactant adsorption capacity and thus prolong the lifetime of (CH_3_SO_3_)_2_Sn/AC. This finding provides a route to deeply develop the Sn-based catalysts in the hydrochlorination of acetylene.
